# Supplementary figures and images for: An observational analysis of the trope “A p-value of < 0.05 was considered statistically significant” and other cut-and-paste statistical methods
Source: PLoS One. 2022 Mar 9;17(3):e0264360. doi: 10.1371/journal.pone.0264360 (PMC8906599; doi:10.1371/journal.pone.0264360)

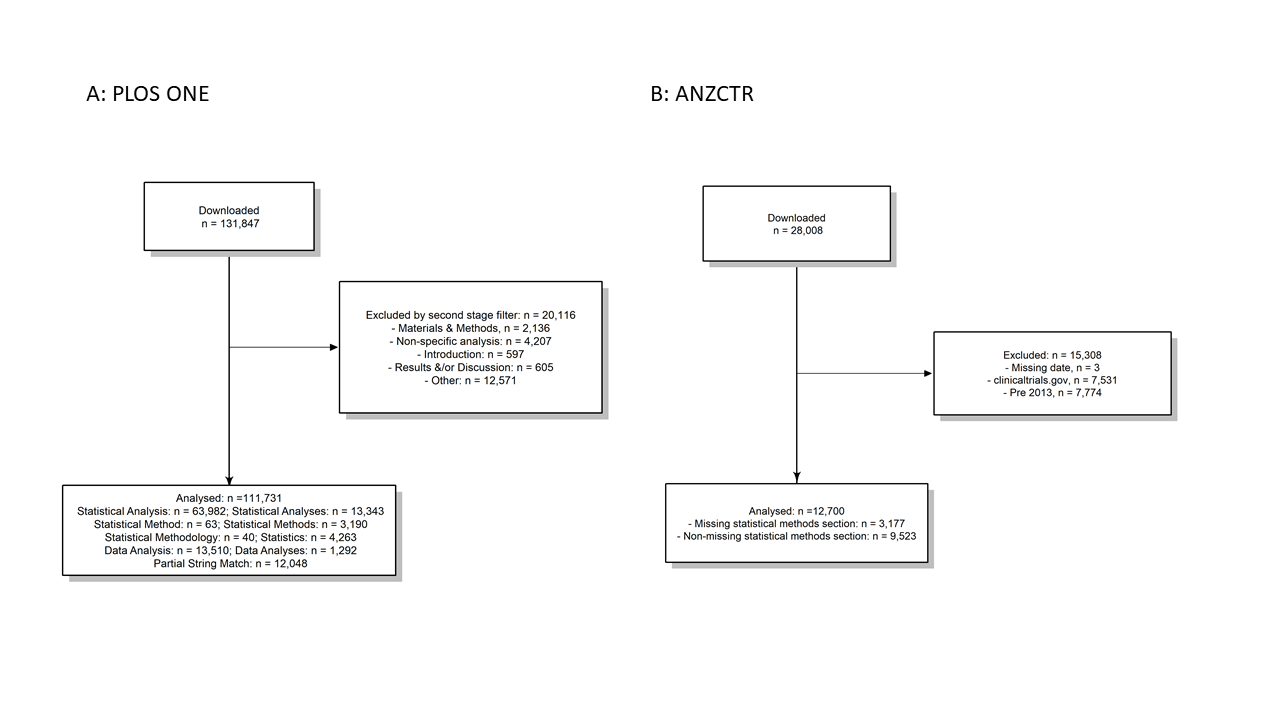

Supplement: S1 Fig — A: PLOS ONE; B: ANZCTR. For (A), “Non-specific analysis” refers to studies where the use of statistical methods could not be determined by on section headings; e.g., “Microarray analysis”. (TIF) [file pone.0264360.s001.tif]

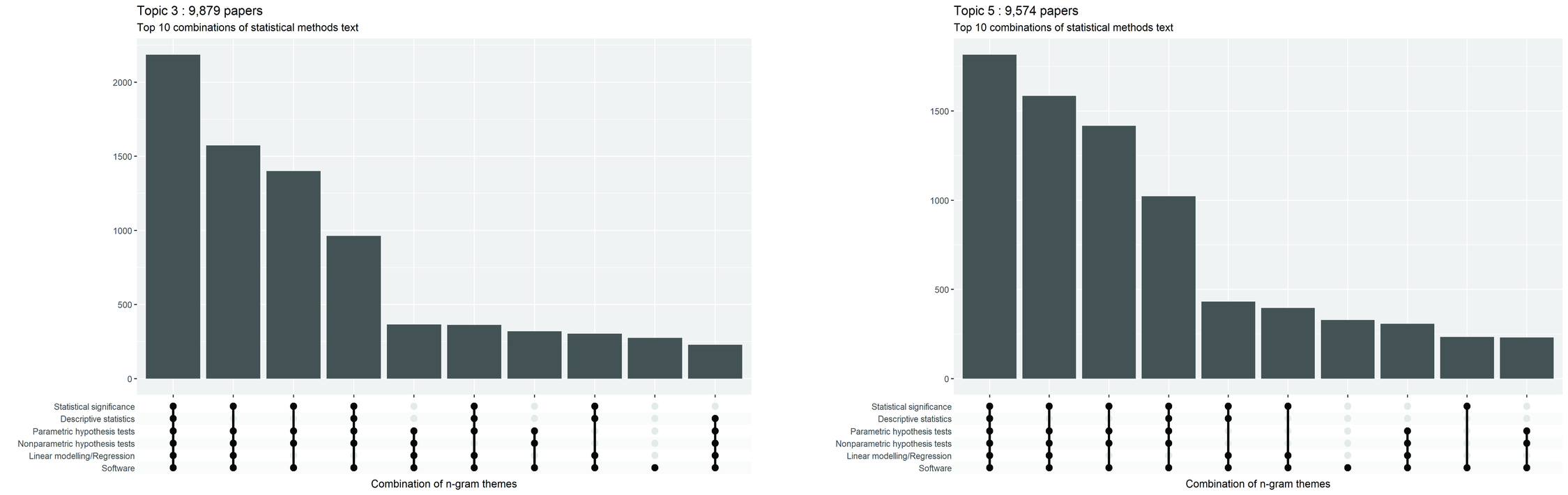

Supplement: S2 Fig — General themes for statistical methods were based on targeted word searches and categorized into statistical significance, descriptive statistics, parametric hypothesis tests, nonparametric hypothesis tests, linear modelling/regression and software. The most frequent combinations of themes are given on the x-axis, with the corresponding number of studies on the y-axis. (TIF) [file pone.0264360.s002.tif]

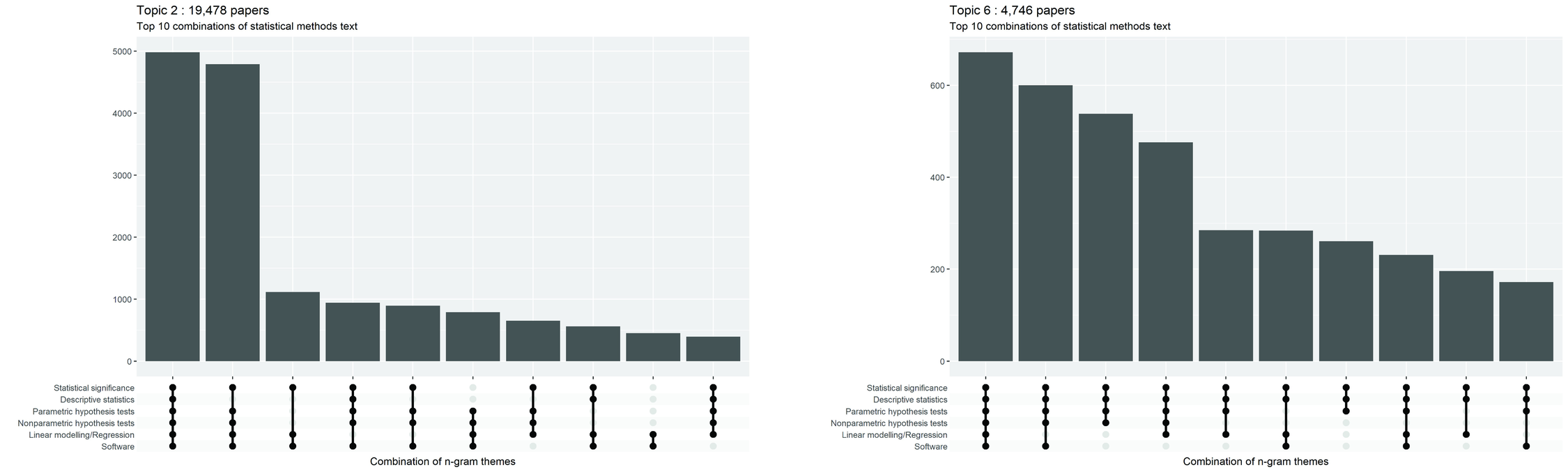

Supplement: S3 Fig — General themes for statistical methods were based on targeted word searches and categorized into statistical significance, descriptive statistics, parametric hypothesis tests, nonparametric hypothesis tests, linear modelling/regression and software. The most frequent combinations of themes are given on the x-axis, with the corresponding number of studies on the y-axis. (TIF) [file pone.0264360.s003.tif]

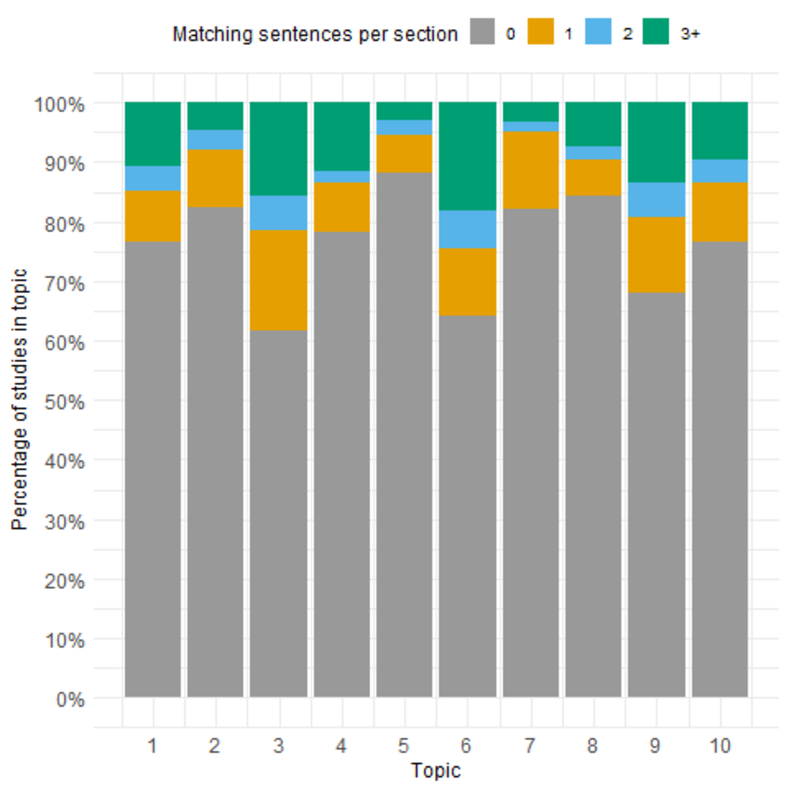

Supplement: S4 Fig — A match was defined any pair of sentences between ANZCTR studies with a Jaccard score equal to 0.9 or higher. (TIF) [file pone.0264360.s004.tif]

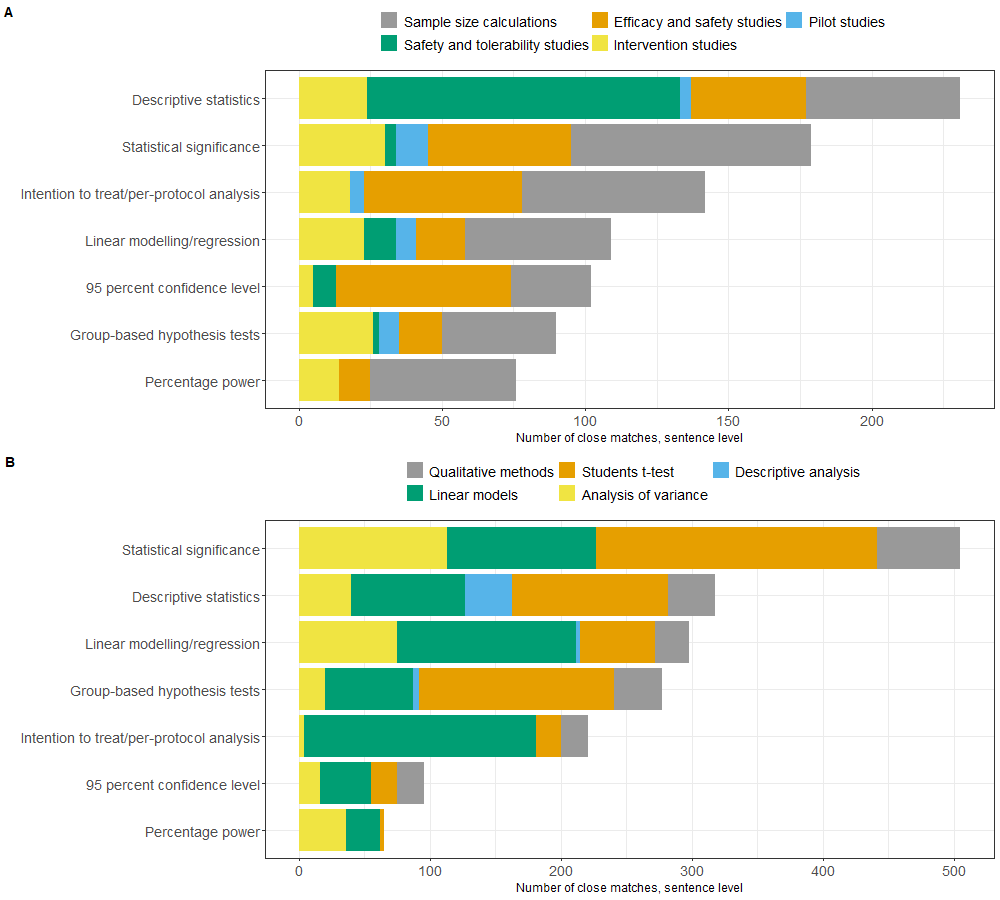

Supplement: S5 Fig — A close match was defined any pair of sentences between ANZCTR studies with a Jaccard score equal to 0.9 or higher. (TIF) [file pone.0264360.s005.tif]
